# The interplay of drug therapeutics and immune responses to SARS-CoV-2

**DOI:** 10.1038/s41423-023-01098-7

**Published:** 2023-11-14

**Authors:** Valeria Fumagalli, Matteo Iannacone

**Affiliations:** 1 Division of Immunology, Transplantation, and Infectious Diseases, IRCCS San Raffaele Scientific Institute, Milan, Italy; 2 Vita-Salute San Raffaele University, Milan, Italy; 3 Experimental Imaging Centre, IRCCS San Raffaele Scientific Institute, Milan, Italy

**Keywords:** SARS-CoV-2, Nirmatrelvir, Bisphosphonates, Adaptive immunity, Drug repurposing, Viral infection, Adaptive immunity, Antimicrobial responses

## Abstract

The SARS-CoV-2 pandemic has necessitated rapid therapeutic and preventative responses. While vaccines form the frontline of defense, antiviral treatments such as nirmatrelvir have emerged as vital adjunctive measures, particularly for those unable or unwilling to be vaccinated. This review delves into the potential influence of nirmatrelvir on enduring immunity. In parallel, the potential of drug repurposing is explored, with bisphosphonates being examined for their possible effects against COVID-19 due to their immunomodulatory properties. The importance of rigorous clinical trials and careful interpretation of preliminary data is emphasized.

## Introduction

The SARS-CoV-2 pandemic has accentuated the urgency for multifarious interventions. Although global vaccination drives have surged, the role of antiviral treatments remains pivotal, especially for select demographics. Nirmatrelvir, an antiviral of interest, offers therapeutic benefits, but potential repercussions on SARS-CoV-2-specific adaptive immune responses are emerging. Concurrently, the strategy of drug repurposing represents an important path to quickly identify potential treatments along with de novo drug development. Within this sphere, bisphosphonates, traditionally used for bone disorders, are being assessed for potential efficacy against COVID-19. This article aims to delineate the therapeutic implications of nirmatrelvir and offer insights into the burgeoning domain of drug repurposing, emphasizing the preliminary nature of data on bisphosphonates.

## Potential negative impact of nirmatrelvir treatment on SARS-CoV-2-specific adaptive immune responses

The SARS-CoV-2 pandemic has necessitated a multifaceted approach to reduce its impact. Although vaccines are the leading frontline defense, antiviral treatments have gained prominence as supportive strategies, especially for those unable or unwilling to be vaccinated. One such antiviral, nirmatrelvir, an orally available Mpro inhibitor [1], is part of the Paxlovid treatment combination known for its effectiveness against COVID-19. However, while its immediate therapeutic impacts are well documented [2–4], its effects on the longer-term adaptive immune response to SARS-CoV-2 have not been extensively explored.

SARS-CoV-2 has an RNA genome encoding two polyproteins, pp1a and pp1ab, and four structural proteins [5]. These polyproteins undergo cleavage to facilitate viral replication, a process involving the 3-chymotrypsin-like cysteine protease (3CLpro) [6, 7]. It is this protease that nirmatrelvir targets, leading to its antiviral effect. However, the impact of nirmatrelvir treatment on the development of SARS-CoV-2-specific adaptive immune responses is uncertain.

**Fig. 1:**
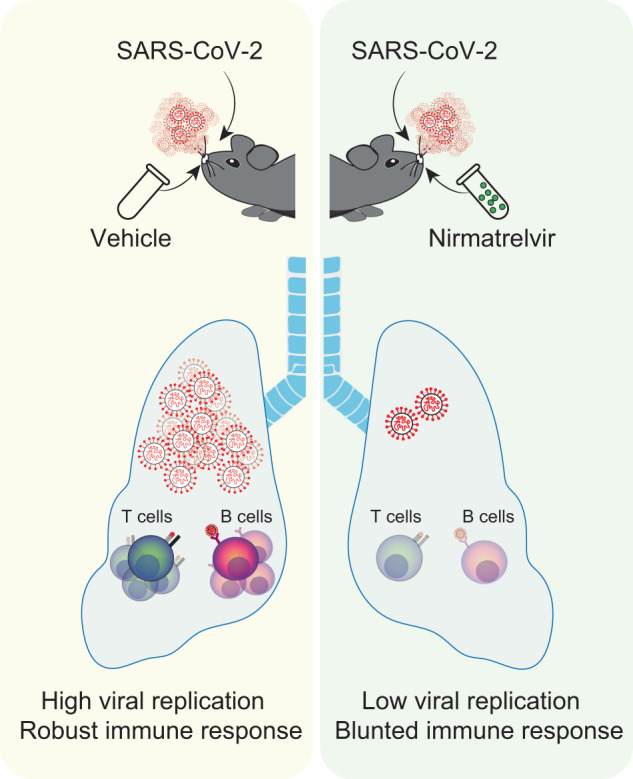
Potential negative impact of nirmatrelvir treatment on SARS-CoV-2-specific adaptive immune responses

Recent studies have provided novel insights into the potential effects of nirmatrelvir on adaptive immunity. Using mouse models, it was observed that administration of nirmatrelvir blunted the development of SARS-CoV-2-specific antibodies and T-cell responses [8] (Fig. 1). This was further corroborated by the observation that upon secondary SARS-CoV-2 exposure, these mice exhibited reduced recruitment of memory T and B cells [8].

The implication is clear: while nirmatrelvir is adept at reducing viral titers and preventing severe disease, it may have unintended consequences on the body’s long-term ability to recognize and combat the virus.

The precise mechanism underlying these findings remains speculative. A plausible explanation might be that nirmatrelvir limits the exposure of the immune system to the viral antigen, thereby compromising the maturation and memory acquisition processes of naïve T and B cells. It is important to contextualize these findings by comparing them with other microbial treatments. For instance, early treatment of *Listeria monocytogenes*-infected mice with amoxicillin does not impair T-cell responses [9–11]. Similarly, administering monoclonal antibody therapy (i.e., bamlanivimab) during acute COVID-19 infection does not hinder the quantity or quality of antigen-specific T-cell responses ~1 month following treatment [12].

This suggests that the impact of antiviral or antimicrobial treatment on adaptive immunity may depend not only on pathogen replication but also on the intricacies of antigen expression, presentation, and innate immunity activation.

The clinical evidence advocating nirmatrelvir for preventing severe COVID-19, especially in high-risk populations, remains robust [2]. However, the potential ramifications for adaptive immunity cannot be overlooked. It becomes crucial to discern whether such effects are exclusive to nirmatrelvir or a broader characteristic of antiviral agents targeting SARS-CoV-2.

Moreover, while mouse studies might not directly reflect human outcomes, these findings underscore the potential side effects of therapeutic interventions. They call for a meticulous reassessment and understanding to optimize treatment strategies, ensuring a balance between immediate therapeutic needs and long-term immune health, for instance, by combining antiviral treatment with active immunization.

The fight against SARS-CoV-2 involves a delicate balance of immediate treatment and ensuring long-term immunity. The findings from recent studies of nirmatrelvir underscore this challenge, emphasizing the need for comprehensive strategies that address both therapeutic and immunological facets of the disease. As we navigate this complex landscape, continued research is paramount to refine our approaches and deliver holistic patient care.

## Repurposing drugs to modulate antiviral immune responses against SARS-CoV-2: The case for bisphosphonates

In the crucible of the COVID-19 pandemic, the international scientific community has ardently pursued the repurposing of existing drugs as potential therapeutic options for viral onslaught. Drug repurposing, the strategy of evaluating a drug already proven safe and effective for a different clinical indication, offers a swift alternative to traditional drug discovery routes. By harnessing both in silico and in vitro analyses, a plethora of drugs have been postulated to show potential efficacy against COVID-19 either through direct antiviral mechanisms or indirect pathways [13]. Furthermore, another intriguing avenue is the exploration of agents that either enhance or modulate the body’s antiviral immune responses against SARS-CoV-2. This could ideally lead to a reduction in clinical symptoms or a retardation in disease progression. The essence remains, however, that it is paramount to test the safety and efficacy of each drug candidate rigorously through randomized clinical trials. Observational studies play a pivotal role in this journey, as they shed light on potential drug candidates by comparing the incidence or severity of the disease in users of a particular drug to a parallel cohort of nonusers [13].

A recent study by von Andrian et al. [14] has drawn attention to bisphosphonates—a well-established class of small-molecule drugs renowned for their ability to inhibit osteoclast-mediated bone resorption [15]. Predominantly prescribed to address conditions such as osteoporosis, Paget’s disease, and malignancy-induced hypercalcemia, bisphosphonates are also employed as an adjuvant therapeutic strategy in breast cancer [16]. Based on their chemical composition, bisphosphonates can be categorized into two main subclasses: nitrogen-containing bisphosphonates (amino-bisphosphonates) and their nonnitrogenous counterparts (non-amino-bisphosphonates) [17]. Although both types are taken up by bone, their molecular mechanisms of action on osteoclasts diverge considerably [18].

Beyond their osteoclastic interactions, bisphosphonates have garnered attention for their multifaceted immunomodulatory effects, propelling them into the spotlight as prospective repurposed agents against COVID-19 [19]. A noteworthy observation is the capability of amino-bisphosphonates to modulate various immune cell types, including human γδT cells [20–22], neutrophils [23], monocytes [24], and macrophages [25, 26]. They can also influence the antigen-presenting ability of dendritic cells [27]. Animal studies have unveiled the potent adjuvant activity of both amino and non-amino bisphosphonates, which can augment antibody and T-cell responses to viral antigens [28]. Moreover, observational studies have highlighted a diminished in-hospital mortality rate for critically ill patients [29] and a marked decrease in the incidence of pneumonia and related mortality in individuals treated with amino-bisphosphonates as opposed to controls [30]. Such compelling clinical outcomes, when combined with their global availability, cost-effectiveness, ease of administration, and well-documented safety profiles across adult [31] and pediatric [32, 33] demographics, position bisphosphonates as formidable contenders in the repurposed drug armamentarium against COVID-19.

With these considerations in the foreground, a meticulous analysis was conducted on a comprehensive U.S. health insurance claims database [14]. The aim was to discern any association between prior bisphosphonate administration and subsequent variations in the incidence or severity of COVID-19 outcomes. A specific focus was on the correlation between bisphosphonate consumption and indices such as COVID-19 hospitalizations, diagnosis, and testing for SARS-CoV-2, especially during the early stages when testing was less accessible [14]. The data spanned from March 1, 2020, to June 30, 2020, encapsulating the initial wave of the pandemic in the U.S., well before the introduction of potential mitigating interventions such as vaccinations or other effective treatments [14].

**Fig. 2:**
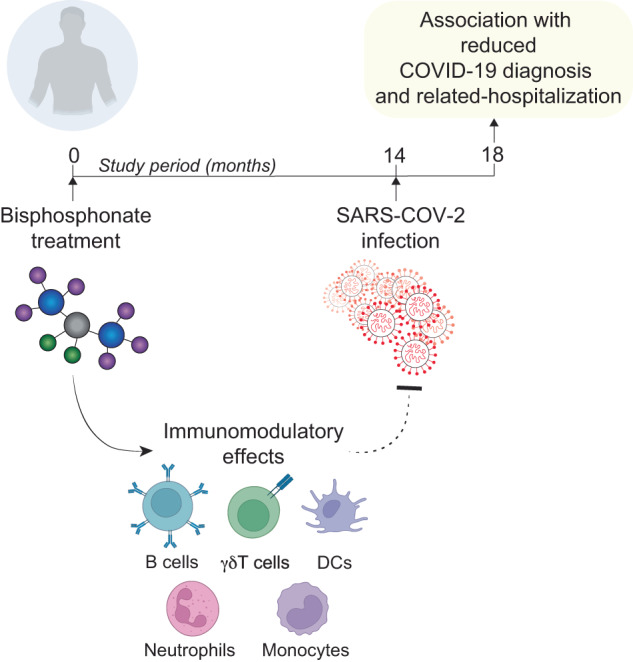
Repurposing drugs to modulate antiviral immune responses against SARS-CoV-2: The case for bisphosphonates

The outcomes were illuminating. It was discerned that individuals who had consumed amino-bisphosphonates manifested a three- to fivefold reduction in the incidence of events such as SARS-CoV-2 testing, a confirmed COVID-19 diagnosis, and related hospital admissions [14] (Fig. 2). This substantial differential remained consistent across various comparisons—whether contrasting bisphosphonate users with nonusers in a matched general cohort, comparing them to individuals taking other bone antiresorptive treatments, or even when the latter cohort was further narrowed down to female osteoporosis patients stratified by other demographic or clinical variables [14]. While retrospective studies inherently possess limitations due to potential uncontrolled confounders, the robustness and consistency of these findings underscore the imperative for additional prospective investigations to better understand the potential immunomodulatory attributes of bisphosphonates in this context.

In conclusion, despite the inherent challenges and caveats associated with retrospective analyses, the correlation between bisphosphonate administration and a reduced likelihood of adverse COVID-19 outcomes remains tenaciously consistent across diverse subgroups—be it age, sex, or geographical location [14]. Such compelling evidence fervently advocates the consideration of bisphosphonates as potential prophylactic agents for those vulnerable to SARS-CoV-2 infection [14]. However, the emphasis remains on the necessity for rigorously conducted prospective clinical studies to unequivocally establish the direct benefits of bisphosphonates, especially in patient subsets where bisphosphonates are not customarily prescribed.

## Conclusion

In confronting the intricate challenges posed by the SARS-CoV-2 pandemic, the combined strategies of direct antiviral interventions and drug repurposing stand as pivotal frontiers. Nirmatrelvir’s therapeutic prowess and its prospective impact on long-term immunity necessitate continuous evaluation. On another front, the repurposing of agents such as bisphosphonates, although promising, stands in its nascent stages, underscoring the dire need for meticulous clinical investigations. As we forge ahead, the emphasis must rest on blending immediate therapeutic solutions with unyielding scientific inquiry, ensuring that we traverse this health conundrum with efficacy and foresight.

While the focus of this review centers on the therapeutic implications of nirmatrelvir and the potential repurposing of bisphosphonates, it is imperative to acknowledge the broader landscape of approved COVID-19 therapeutics. The FDA and the EUA have greenlit several therapeutics, encompassing antivirals such as remdesivir and molnupiravir, anti-inflammatory treatments including dexamethasone, baricitinib, and tocilizumab and various monoclonal antibodies. Each of these therapeutics may influence antiviral immunity. For instance, nonsteroidal anti-inflammatory drugs have been found to impair the neutralizing antibody response to SARS-CoV-2 in mice [34]. Several monoclonal antibodies, with half-lives between 18 and 25 days, neutralize SARS-CoV-2 and serve both preventive and therapeutic roles. Some studies indicate that these antibodies, when administered early, might suppress the primary IgM antiviral response [35]. Proposed mechanisms behind this interference include epitope masking and viral antigen load reduction [35]. However, other research suggests that recipients of these antibodies can still elicit robust adaptive immunity against SARS-CoV-2 [12, 36–38]. Notably, while early guidance from the CDC and WHO advised a 90-day gap post monoclonal antibody therapy before COVID-19 vaccination [39], current perspectives often recommend immediate vaccination considering the benefits.

It is worth noting that while these treatments have demonstrated promise and garnered regulatory approval, a comprehensive assessment of their long-term implications on antiviral immunity is still underway. Such evaluations are crucial for understanding the full spectrum of effects these drugs might have on the host immune response. This review has chosen not to delve deeply into these therapeutics, as more comprehensive data are needed before drawing definitive conclusions on their long-term impact on antiviral immunity.
